# Global scientific trends on the immunomodulation of mesenchymal stem cells in the 21st century: A bibliometric and visualized analysis

**DOI:** 10.3389/fimmu.2022.984984

**Published:** 2022-08-24

**Authors:** Zhongqing Wang, Yuqiang Sun, Rou Shen, Xia Tang, Yingxin Xu, Ye Zhang, Yao Liu

**Affiliations:** ^1^ Department of Information Center, The First Hospital of China Medical University, Shenyang, China; ^2^ Department of Emergency, The First Hospital of China Medical University, Shenyang, China; ^3^ Department of Pediatric Dentistry, School and Hospital of Stomatology, China Medical University, Shenyang, China; ^4^ Liaoning Provincial Key Laboratory of Oral Diseases, Shenyang, China

**Keywords:** mesenchymal stem cells, immunomodulation, immunosuppression, immunotherapy, bibliometric analysis, immunoregulation

## Abstract

**Background:**

Since the discovery of the immunomodulatory functions of mesenchymal stem cells (MSCs), their application in immunomodulation has attracted considerable attention, and an increasing number of studies have been conducted worldwide. Our research aimed to investigate the global status and trends in this field.

**Methods:**

Publications on the immunomodulatory functions of MSCs from 1 January 2000 to 7 March 2022 were retrieved from the Web of Science Core Collection. The data were studied and indexed using the bibliometric methodology. Visualization analysis, co-authorship, co-occurrence analysis, and publication trends in MSC immunomodulation were conducted using the VOSviewer software.

**Results:**

In total, 4,227 papers were included in the study. The number of publications and research interests has significantly increased globally. China published the highest number of related articles, while the US published articles with the highest number of citations. *Stem Cell Research & Therapy* had the highest number of publications. Sun Yat-sen University, Shanghai Jiao Tong University, Harvard University, and Seoul National University were the most contributive institutions. Furthermore, the studies were divided into four research hotspots for MSC immunomodulation: MSC immunomodulation in regenerative medicine, the effects and mechanisms of MSC immunomodulation, MSC therapy for immune diseases, and the cell source of MSCs.

**Conclusion:**

This study indicates that the number of publications on MSC immunomodulation will increase in the future, and MSC immunomodulation mechanisms and clinical applications of MSC immunotherapy should be the next hotspots in this research field.

## Introduction

Mesenchymal stem cells (MSCs) were first identified and isolated from the bone marrow as an adherent fibroblast-like population (1). Although bone marrow MSCs (BMMSCs) are recognized as classic MSCs, non-marrow-derived MSCs are identified in other tissues, including the placenta, umbilical cord blood, adipose tissue, fetal liver, muscle, lung, dermis, amniotic fluid, orofacial tissue and so on (2). MSCs are rapidly adherent to tissue culture vessels, with multiple differentiation potentials, positive expression of CD73, CD90, CD105, CD44 and Sca1, and negative expression of hematopoietic markers, CD45, CD34, CD14, CD11b, CD79a, and human leukocyte antigen DR isotype (HLA-DR) (3, 4). Based on their key properties of self-renewal and differentiation into mesenchymal and non-mesenchymal lineages, MSCs have been widely used therapeutically as a promising cell source for regenerative medicine.

Emerging evidence indicates a unique immunoregulatory effect of MSCs on several subsets of immune cells, including T lymphocytes, B lymphocytes, natural killer cells, and dendritic cells (5, 6). *In vitro*, MSCs induce cell apoptosis of T lymphocytes and significantly inhibit immune cell proliferation and pro-inflammatory cytokine production (7–9). Moreover, MSCs reduce antibody production of B lymphocytes and suppress the generation and function of antigen-presenting cells (10–12). From 2000 to the present, there have been numerous *in vivo* studies and clinical trials on the therapeutic effects of MSCs immunomodulation (13). These studies indicated that systemic transplantation of MSCs has been successfully used to treat various autoimmune diseases, including graft-versus-host disease (GVHD), systemic lupus erythematosus (SLE), rheumatoid arthritis (RA), and inflammatory bowel disease (IBD) (14–17). MSCs regulate the local immune environment and provide suitable “soil” for tissue regeneration (18, 19). Multiple therapeutic mechanisms contribute to MSC-based cell therapies, including paracrine secretion and interactions between MSCs and immune cells (20). The immunomodulatory effect of MSCs renders them significant for translational applications in tissue regeneration and autoimmune conditions.

Bibliometric analysis is a mathematical and statistical method for assessing the quantitative fluctuations, distributions, and change rules of published literature (21). It enables the quantitative measurement of profile distribution and the relationship and clustering of studies. It provides objective scientific indicators for evaluating research trends, developing guidelines, and treating diseases (22). Bibliometrics has been used in various fields of medicine, including stem cell fields, such as stem cells in cardiovascular diseases (23) and osteoarthritis (24). MSCs have received significant attention in immunotherapy (25). However, to the best of our knowledge, quantitative and qualitative analyses of this research topic have not been reported. Therefore, this study analyzed publications on MSC immunomodulation in the 21st century, highlighting the current status of global research, evidence of trends, and topics requiring further exploration.

## Materials and methods

### Date source and search strategy

Bibliometric analysis was ultimately performed online using the Web of Science (WoS) database, given it is a commonly widely used database with rich bibliometric indicators for analysis. This analysis was performed on a single day to avoid daily updating bias since the database remains open.

**Figure 1:**
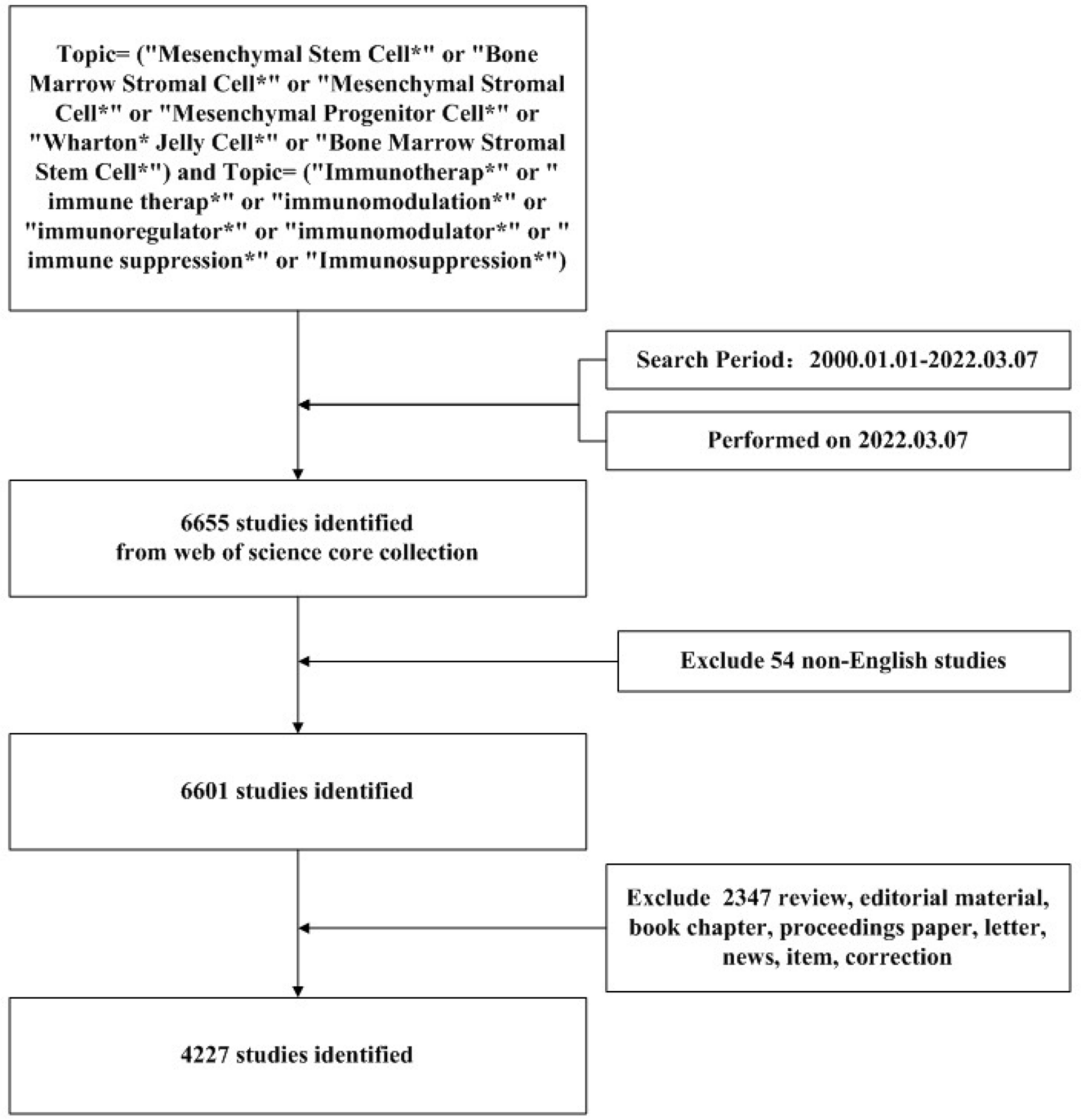
Search strategy for retrieving publications on MSC immunomodulation. The search formula was set as follows: TS = “Mesenchymal Stem Cell*” OR “Bone Marrow Stromal Cell*” OR “Mesenchymal Stromal Cell*” OR “Mesenchymal Progenitor Cell*” OR “Wharton* Jelly Cell*” OR “Bone Marrow Stromal Stem Cell*” AND TS = (“Immunotherap*” OR “immune therap*” OR “immunomodulation*” OR “immunoregulator*” OR “immunomodulator*” OR “immune suppression*” OR “Immunosuppression*”). The publication date was from 1 January 2000, to 7 March 2022. The 6,655 studies were identified from the Web of Science Core Collection (WoSCC), and 54 non-English studies were excluded. The search strategy identified 4,227 studies and excluded 2347 non-articles.

The search strategy was shown in **Figure 1**. Publications in research of the immunomodulation of MSCs were searched for in Web of Science Core Collection (WoSCC) on 7 March 2022. The search formula was set as follows: TS= (“Mesenchymal Stem Cell*” OR “Bone Marrow Stromal Cell*” OR “Mesenchymal Stromal Cell*” OR “Mesenchymal Progenitor Cell*” OR “Wharton* Jelly Cell*” OR “Bone Marrow Stromal Stem Cell*”) AND TS= (“Immunotherap*” OR “immune therap*” OR “immunomodulation*” OR “immunoregulator*” OR “immunomodulator*” OR “immune suppression*” OR “Immunosuppression*”). The time period of article publication was from 1 January 2000 to 7 March 2022. For manuscript types, only peer-reviewed articles in English were included. The full records of each publication, including title, year of publication, authors’ names, nationalities, affiliations, name of the journal, keywords, and abstract, were downloaded from the WoS database and imported into Microsoft Excel 2016.

### Visualized analysis

VOSviewer is the classic bibliometric analysis software, which was used to perform visualized analysis in this study (26). VOSviewer (version 1.6.18) was used to analyze the co-authorship, co-occurrence, established co-authorship network visualization map, keyword network visualization map, and overlay visualization map. Additionally, a descriptive analysis was also conducted which included publication years, journals, highly-cited papers, countries, institutions and authors.

## Results

### Number of global publications

**Figure 2:**
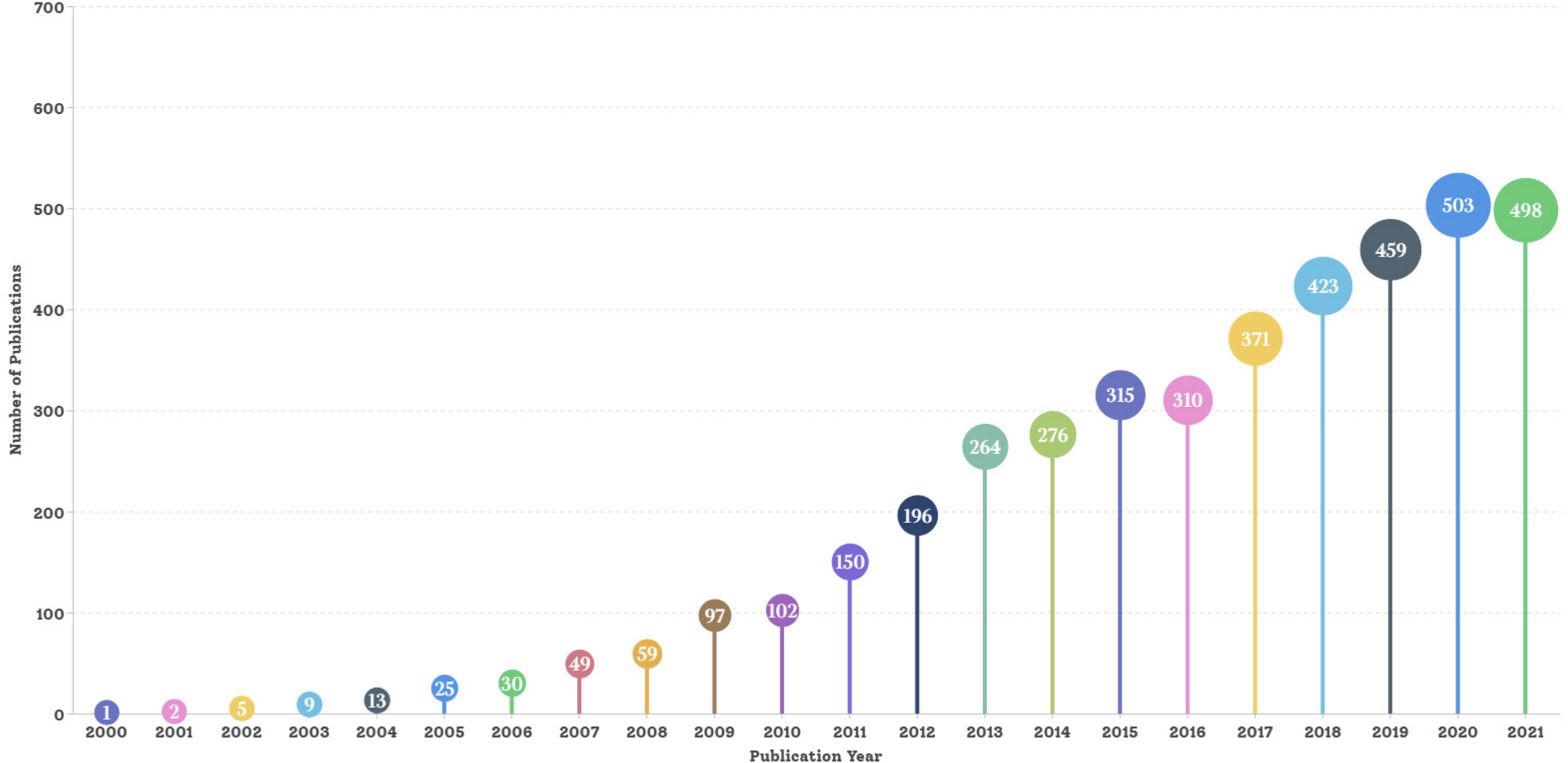
Trends in number of global publications on MSC immunomodulation 2000−2022. A total of 4,227 articles from 2000 to 2022 met the search criteria. Starting from one article in 2000, the number of articles published in 2010 exceeded 100, with more than 300 articles in 2015 and more than 400 articles in 2018 until 2021.

A total of 4,227 articles from 2000 to 2022 met the search criteria (excluding 54 non-English and 2,347 non-article publications). As shown in **Figure 2**, there was a significant upward trend in global publications on MSC immunomodulation per year (publications in 2022 were excluded because the statistical data were not completed as of 7 March 2022). Global publications were divided into three stages, according to the development of this research field. The “cultivation period” was from 2000 to 2009, with a small number of publications per year, ranging from several to dozens. From 2010 to 2016 was the “unk development period”, with the number of publications exceeding 100 in 2010 and 300 in 2015 (3.04 times that in 2010). From 2017 to 2021 was the “unk boom period”, with 2,254 articles, which accounted for 53.32% of all publications. The number of publications increased annually to a peak in 2020 (503 publications, 11.90%).

### Contributions of countries

**Figure 3:**
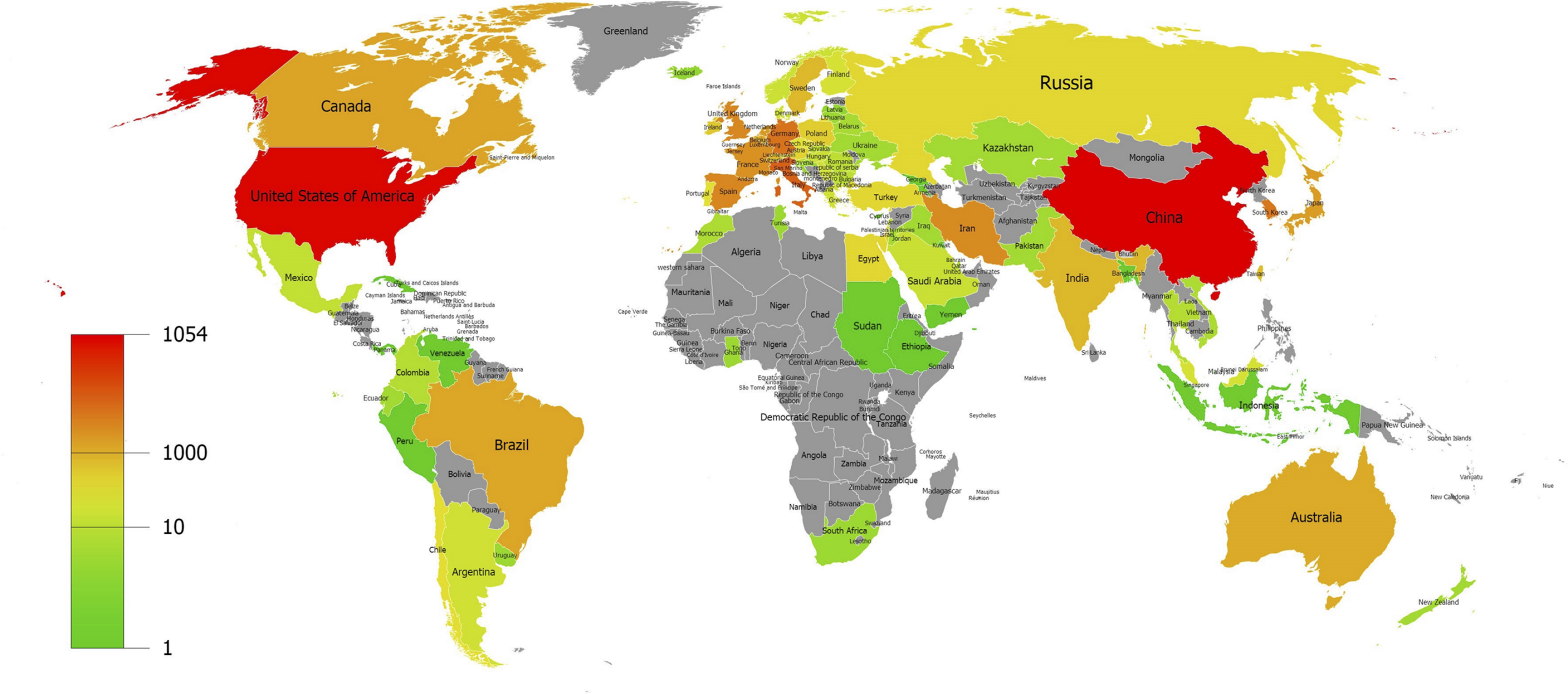
Geographic distribution of global publications on MSC immunomodulation. World map showing the distribution of global publications on MSCs immunomodulation.

A total of 82 countries and regions contributed to the field of MSC immunomodulation, as shown by the geographic distribution of global publications in **Figure 3**. The countries of published articles were mainly distributed in Asia, led by China, and North America, led by the US. The top ten contributive countries with publications in this research field are shown in **Table 1**. China published the largest number of articles (1,054, 24.93%), followed by the US (1,000, 23.66%) and Italy (342, 8.09%). Publications from the US had the highest citation frequency (53,856 citations), followed by China (30,671 citations), Italy (17,148 citations), and Germany (10,345 citations). Regarding average citations, articles published from France had the highest average citation frequencies (64 citations), indicating that publications from France were of high quality. Additionally, Iran (the average publication year was 2018.14) had the latest publication year among the top ten contributive countries.

**Table 1:** Top 10 contributive countries with publications on the immunomodulation of MSCs.

Rank	Country and Region	Documents	Citations	Average citations	Average publication year
1	China	1054	30671	29	2016.95
2	United States	1000	53856	54	2015.81
3	Italy	342	17148	50	2015.81
4	Germany	266	10345	39	2016.15
5	South Korea	251	6417	26	2016.54
6	Spain	197	6526	33	2016.93
7	United Kingdom	195	7437	38	2016.59
8	Iran	185	2626	14	2018.14
9	France	168	10720	64	2015.42
10	Netherlands	150	8463	56	2015.48

### Contributive analysis of journals

4227 articles on the immunomodulation of MSCs were published by 884 journals in total. There were 1,168 articles (27.63%) published in the top 10 journals (**Table 2**). Stem Cell Research & Therapy (IF=6.832, 2020) published the most relative articles (240 publications). Stem Cells and Development (IF=3.272, 2020) ranked second with 123 publications, followed by PLOS ONE (IF=3.240, 2020) with 119 publications. The Publications on the immunomodulation of MSCs in Stem Cells (IF=6.277, 2020) had the highest total citations (12808 times) and average citations (110 times). About the latest average publication year in the top 10 journals, in Stem Cell Research & Therapy was 2018.32 and in Frontiers in Immunology was 2018.30, which indicating these two journals published lots of articles in recent years.

**Table 2:** Top 10 journals contributed to publications on the immunomodulation of MSCs.

Rank	Journal	Impact factor(2020)	Counts	Citations	Average citations	Average publication year
1	Stem Cell Research & Therapy	6.832	240	4819	20	2018.32
2	Stem Cells and Development	3.272	123	4418	36	2015.00
3	PLOS ONE	3.240	119	5317	45	2014.21
4	Cytotherapy	5.414	117	4160	36	2014.72
5	Stem Cells	6.277	116	12808	110	2014.13
6	Stem Cells Translational Medicine	6.940	113	4338	38	2016.99
7	Scientific Reports	4.379	94	2539	27	2017.49
8	Stem Cells International	5.443	83	1118	13	2017.42
9	Frontiers in Immunology	7.561	82	2011	25	2018.30
10	Cell Transplantation	4.064	81	2922	36	2014.28

### Highly cited literature analysis

The top ten articles with the highest citations on MSC immunomodulation are shown in **Table 3**. Among them, five articles, including the article with the highest citation frequency (3,591 citations), were published in *Blood*, *Cell Stem Cell*, *Transplantation*, *Experimental Hematology* and *Stem Cells* respectively. The article with the highest citation frequency, “Human mesenchymal stem cells modulate allogeneic immune cell responses”, was published by Aggarwal *et al.* in 2005. The latest publication with a high number of citations (1,037 citations) was “The MSC: An Injury Drugstore”, published in *Cell Stem Cell* in 2011.

**Table 3:** Top 10 articles on the immunomodulation of MSCs with the high citations.

Rank	Year	First Author	Title	Source	Citations
1	2005	Aggarwal S	Human mesenchymal stem cells modulate allogeneic immune cell responses.	Blood	3591
2	2002	Bartholomew A	Mesenchymal stem cells suppress lymphocyte proliferation *in vitro* and prolong skin graft survival *in vivo*.	Experimental Hematology	1950
3	2008	Ren G	Mesenchymal stem cell-mediated immunosuppression occurs *via* concerted action of chemokines and nitric oxide.	Cell Stem Cell	1431
4	2003	Tse WT	Suppression of allogeneic T-cell proliferation by human marrow stromal cells: implications in transplantation.	Transplantation	1293
5	2005	Zappia E	Mesenchymal stem cells ameliorate experimental autoimmune encephalomyelitis inducing T-cell anergy.	Blood	1159
6	2011	Caplan AI	The MSC: an injury drugstore.	Cell Stem Cell	1037
7	2006	Krampera M	Role for interferon-gamma in the immunomodulatory activity of human bone marrow mesenchymal stem cells.	Stem Cells	1012
8	2003	Djouad F	Immunosuppressive effect of mesenchymal stem cells favors tumor growth in allogeneic animals.	Blood	1002
9	2006	Ringdén O	Mesenchymal stem cells for treatment of therapy-resistant graft-versus-host disease.	Transplantation	935
10	2005	Beyth S	Human mesenchymal stem cells alter antigen-presenting cell maturation and induce T-cell unresponsiveness.	Blood	864

### Contributive institutions analysis

A total of 4,186 institutions contributed to the research field of MSC immunomodulation. As shown in **Table 4**, the most contributive institution was Sun Yat-sen University (113 publications), followed by Shanghai Jiao Tong University (99 publications), Harvard University (86 publications), Seoul National University (71 publications), and Chinese Academy of Sciences (69 publications). Moreover, among the top ten institutions, Harvard University had the article with the highest total and average citation frequency (8,872 and 103 citations, respectively).

**Table 4:** Publications in the top 10 organizations.

Rank	Institutions	Counts	Citations	Average citations	Average publication year
1	Sun Yat-sen University	113	2869	25	2017.82
2	Shanghai Jiao Tong University	99	3988	40	2017.32
3	Harvard University	86	8872	103	2014.73
4	Seoul Natl University	71	1920	27	2016.32
5	Chinese Academy of Sciences	69	2541	37	2017.75
6	Chinese Academy of Medical Sciences	57	3919	69	2014.28
7	University of Sao Paulo	53	790	15	2017.00
8	Tianjin Medical University	51	1059	21	2015.90
9	Karolinska Institute	47	2647	56	2015.09
10	Fourth Military Medical University	46	1535	33	2015.67

**Figure 4:**
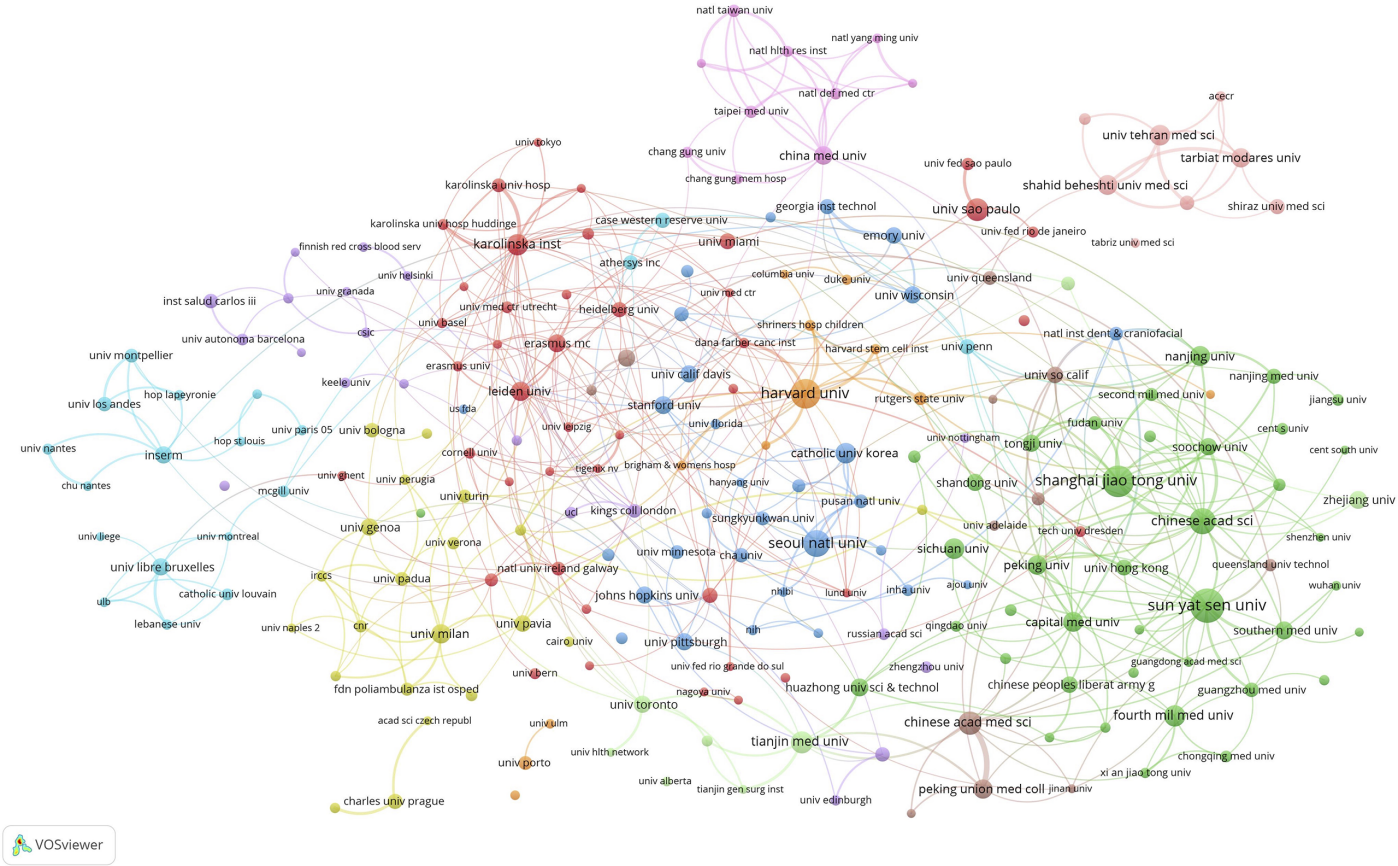
Mapping institutional co-authorship on MSC immunomodulation publications. The size of the points represents the institutional co-authorship frequency. The line between the two points indicates established collaboration between the two institutions. The thickening of the line indicates the collaboration degree between the two institutions.

The minimum number of institutional publications was set as 10, and 224 contributive institutions were selected for institutional co-authorship analysis using the VOSviewer software (**Figure 4**). Among them, 223 institutions formed the largest institutional co-authorship network and were divided into 11 clusters. The largest cluster (red cluster), consisting of 42 institutions, was centered at the University of São Paulo and the Karolinska Institute. The green cluster, consisting of 49 institutions mainly located in China, ranked second and was centered at Sun Yat-sen University, Shanghai Jiao Tong University, and the Chinese Academy of Sciences. The top three institutions with the largest total link strength were Harvard University (total link strength = 51 times), Chinese Academy of Sciences (total link strength = 43 times), and Sun Yat-sen University (total link strength = 34 times), indicating that they were the most contributive institutions.

### Contributive analysis of authors

A total of 24,049 authors have published articles on MSC immunomodulation from 2000 to 2022. The top ten most productive authors are listed in **Table 5**. The two authors with the most publications were Lagneaux Laurence (37 publications) and Najar Mehdi (37 publications). Shi Yufang had the highest citation frequency (4,474 citations). In addition, the average publication year of Hashemi Seyed Mahmoud was 2017.89, which was the latest average publication year among the top ten contributive authors.

**Table 5:** Top 10 contributive authors with publications on the immunomodulation of MSCs.

Rank	Author	Counts	citations	Average citations	Average publication year
1	Lagneaux Laurence	37	1291	35	2015.57
2	Najar Mehdi	37	1247	34	2015.68
3	Hoogduijn Martin J.	31	1738	56	2014.71
4	Parolini Ornella	30	1491	50	2015.43
5	Shi Yufang	29	4474	154	2014.00
6	Hashemi Seyed Mahmoud	28	504	18	2017.89
7	Shi Songtao	26	2737	105	2014.23
8	Jorgensen Christian	23	2459	107	2014.78
9	Bron Dominique	23	1183	51	2014.22
10	Sun Lingyun	22	1530	70	2015.18

**Figure 5:**
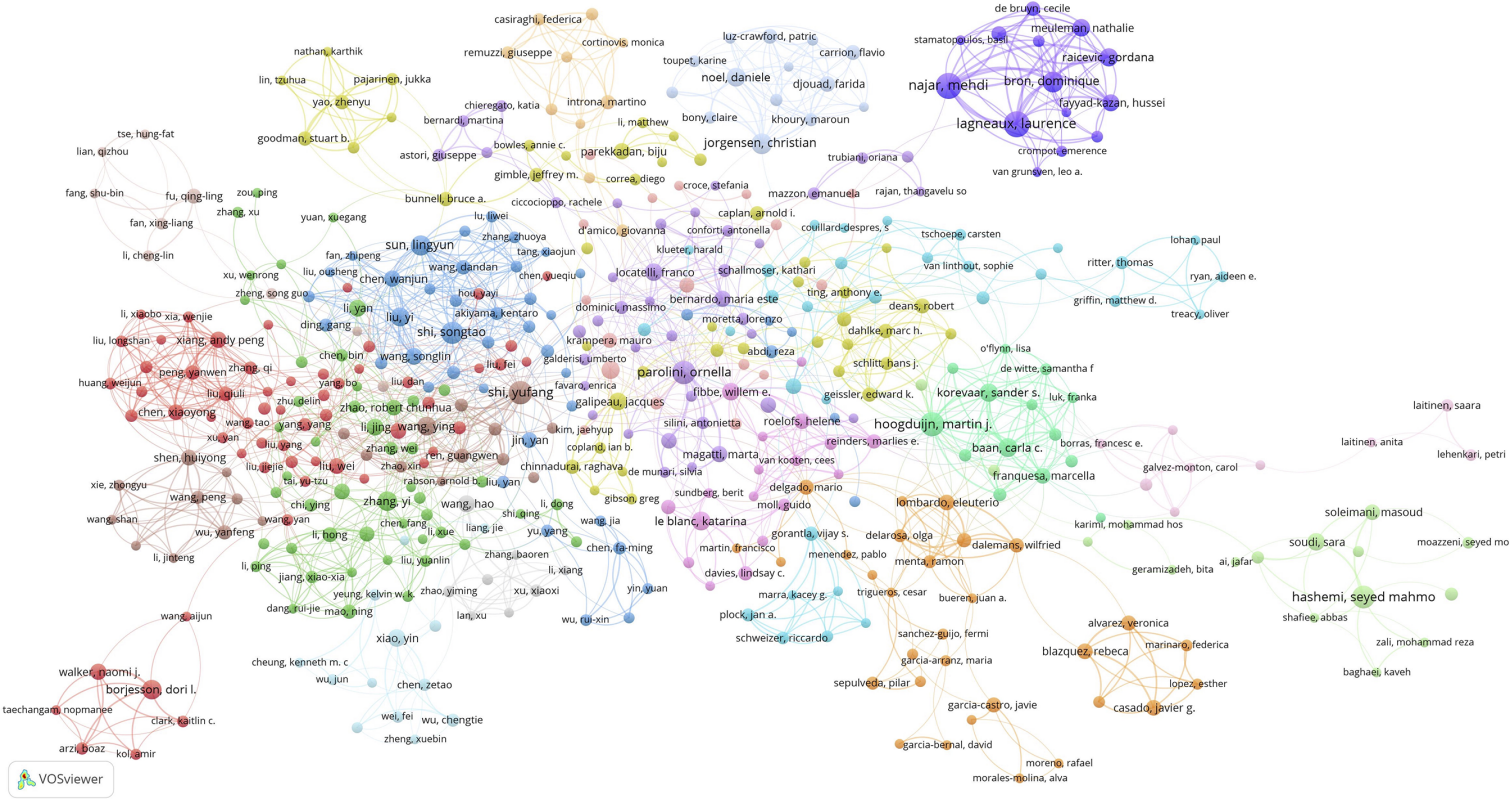
Mapping author co-authorship on MSC immunomodulation publications. The size of the points represents the co-authorship frequency of authors. The line between the two points represents established collaboration between the two authors. The thickening of the line represents the collaboration degree between the two authors.

The minimum number of author publications was set as five, and 648 contributive authors were selected for author co-authorship analysis using the VOSviewer software. In addition, 458 authors formed the largest author co-authorship network, which was divided into 19 clusters (**Figure 5**). The red cluster (58 authors) was the largest co-authorship cluster. The dark blue cluster (14 authors), centered on Lagneaux Laurence, Najar Mehdi, and Bron Dominique, was a close-knit research team. These three authors collaborated several times; for example, Lagneaux Laurence and Najar Mehdi collaborated 35 times, and Lagneaux Laurence and Bron Dominique collaborated 23 times. The top three authors with the largest total link strength were Shi Yufang (total link strength = 39 times), Shi Songtao (total link strength = 35 times), and Hoogduijn Martin (total link strength = 31 times).

### Keyword analysis

**Figure 6:**
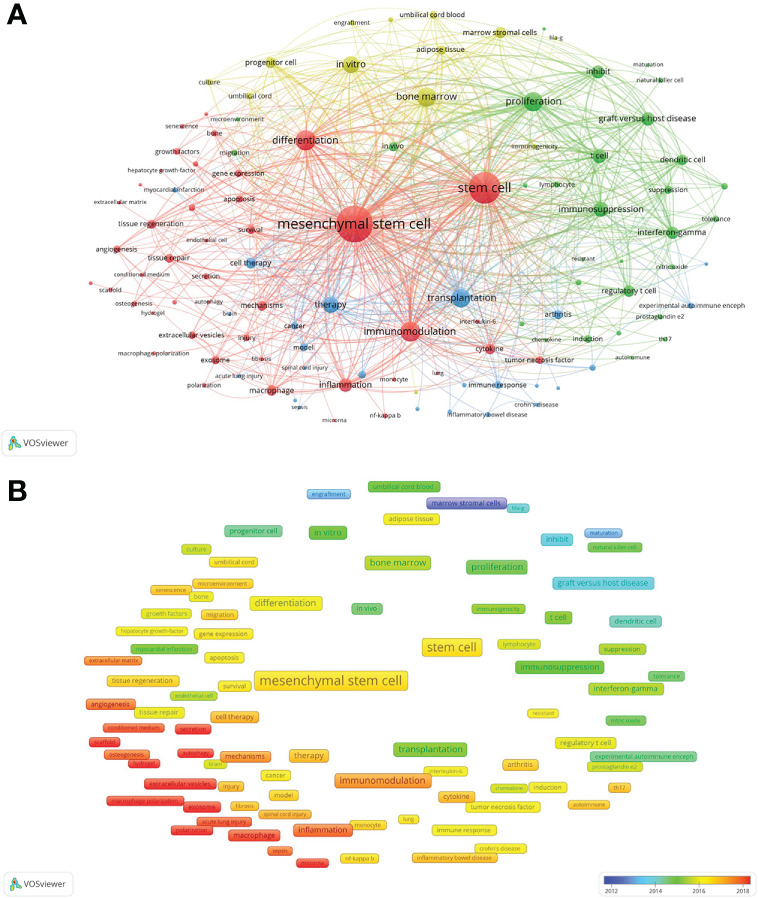
Mapping co-occurrence of high-frequency keywords on MSC immunomodulation publications. **(A)** Mapping of keywords appeared in the publications. **(B)** Distribution of keywords according to the average publication year.

There were 10,599 keywords in the 4,227 publications on MSC immunomodulation. The top ten keywords with the highest frequencies were MSCs (2,567 times), stem cell (1,995 times), differentiation (904 times), immunomodulation (861 times), inflammation (440 times), macrophage (266 times), cytokine (263 times), mechanisms (239 times) and tissue regeneration (202 times). A total of 111 keywords (**Supplementary Table 1**), defined as high-frequency keywords used more than 40 times, were selected for co-occurrence analysis and classified into four clusters using the VOSviewer software. As shown in **Figure 6A**, the largest cluster (red cluster), including 43 keywords, focused on “MSCs immunomodulation in regenerative medicine”. The keywords in the other three clusters concentrated on “the effects and mechanisms of MSCs immunomodulation” (green cluster), “MSC therapy for immune diseases” (blue cluster), and “the cell source of MSCs” (yellow cluster).

A total of 111 high-frequency keywords were color-coded based on the average year of appearance in the publications (**Figure 6B**). The blue color-coded keywords indicated early publications, and the red color-coded keywords indicated the latest publications. The top five latest keywords were exosome (2019.29), extracellular vesicles (2019.15), macrophage polarization (2019.15), polarization (2018.56), and scaffold (2018.45).

## Discussion

Bibliometrics combined with visualization analysis produces an overview of certain research fields of interest, guiding further research. This study was performed to evaluate the contribution of countries, institutions, authors, core literature, and research hotspots and identify the developmental trend of the research field of MSC immunomodulation. The main outcomes of the analysis are discussed in subsequent subsections.

### Trends and status of global publications

Although MSCs were first isolated and characterized in 1974 (27), the immunosuppressive effects of BMMSCs were not reported until 1995 (28). This study demonstrates that there has been a sustained increase in the number of publications on the immunomodulatory properties of MSCs per year. More than half of the related studies were published from 2018 to 2022, indicating increased research interest and a continuous upward trend. We predict that more studies with in-depth knowledge and insightful understanding of MSC immunomodulation will be published subsequently. Moreover, of the total 82 countries that contributed to global publications, China published the most relevant articles (1,054 articles), followed by the US (1,000 articles). Analysis of the citations representing the academic impact and quality of publications indicated that the total citation frequency of the US (53,856 citations) was the highest, much higher than that of China (30,671 citations), indicating that the US could be regarded as the leading and principal contributive country in this research field. These data indicate an assignable imbalance between the number and quality of studies in China.

Considering the quantity and quality of journals, the core publishing journals on MSCs immunomodulation were listed. Authors interested in this field should focus on these journals and consider them when contributing to a relevant paper. Moreover, *Stem Cells* ranked first in total and average citation frequencies, indicating that their publications are of great significance and reference value; *Stem Cell Research & Therapy* ranked first in the average publication years and the number of publications, indicating that it updates publications actively and grasps the frontiers of dynamic research.

The top five productive institutions from China, the US, and Korea were the leading institutions in the field of MSC immunomodulation. Among them, Harvard University in the US produced many high-quality articles corresponding with the top country in citation frequency. This demonstrates that advanced research institutions may be a determining factor at the academic level of a country. Furthermore, a co-authorship analysis was performed to evaluate the collaboration between institutions and authors; a higher total link strength indicated that the research institutions/authors preferred collaboration. The most predominant institutions were Harvard University, Chinese Academy of Sciences, and Sun Yat-sen University. The top three contributive authors were Shi Yufang, Shi Songtao, and Hoogduijn Martin. It was also revealed that the collaborative institutions/authors as the cluster centeres usually belonged to the top contributive section, suggesting that collaboration with other institutions/authors would promote the development of certain scientific fields, and the interest and enthusiasm for studies may also be important subjective factors.

### Research hotspots focus on MSCs immunomodulation

Co-occurrence analysis was applied to determine interests and popular topics in the research field, which will guide researchers in in-depth studies. From the co-occurrence mapping of keywords in the field of MSC immunomodulation, it was revealed that keywords including stem cells, MSC, immunomodulation, and differentiation were conspicuously shown with higher weight in size and relation strength than other keywords. The research hotspots extracted from all included studies were divided into four clusters for different research directions.

#### Red cluster: The immunomodulation of MSCs in regeneration medicine

MSCs participate in the whole tissue regeneration process as the primary stem cell source. The immunoregulatory effects of MSCs are primarily involved in this research direction, supporting their application in regenerative medicine. This cluster suggested that prominent keywords, such as “MSC differentiation”, “tissue inflammation” and “immunomodulation”, were the main research directions. MSCs significantly promote tissue regeneration by regulating the subtype distribution and phenotypes of immune cells, including macrophage polarization (29). Moreover, MSCs immunosuppress inflammatory response in the early phase of tissue injury, resulting in less scarring and tissue regeneration (30). The immunomodulatory properties of MSCs also rely on the paracrine effects, including cytokines and extracellular vesicles (31, 32). Emerging evidence has demonstrated that MSC-derived exosomes have immunomodulatory functions similar to those of donor MSCs (33–35). The immunomodulation of MSC-derived exosomes is hypothesized to be a future research hotspot.

#### Green cluster: The effects and mechanisms of the MSC immunomodulation

Since Hamburger *et al.* first reported that BMMSCs produce immuno-cytokines to contribute to the suppression of hematopoiesis (36), the immunomodulatory effects of MSCs and their underlying mechanisms have been of interest to investigators. In 2005, the first most-cited article innovatively described the phenomenon of MSCs regulating allogeneic immune cells, suggesting that prostaglandin E2 (PGE2) may be an important MSC immunomodulator (5). There were several similar keywords, including “immunosuppression”, “inhibit” and “suppression”, among the more prevalent ones in the literature. These keywords were identified as the principal effects of MSC immunomodulation. Cluster mapping also demonstrated the mechanism of MSC-developed immunosuppressive effects, such as involved “immune cells”, “proliferation” and “inflammatory cytokines”. The investigators reported that MSCs directly induced the apoptosis of T lymphocytes, inhibited the proliferation of immune cells, and reduced cytokine secretion *via* soluble factors and cell-to-cell contact (37, 38), involving the participation of immune cells, including T lymphocytes, regulatory T cells, B cells, and dendritic cells (39–42). TNFα/TNFR2 signaling pathway, as an immune checkpoint axis, plays a critical role in the immunomodulatory effects of MSCs through direct suppression of T cells and indirect induction of active Foxp3^+^ Tregs (43, 44).

#### Blue cluster: MSC therapy for immune diseases

MSCs have quickly attracted considerable attention in the clinical field considering their promising features including regenerative properties and immunomodulatory ability. To 2016, 493 MSC-based clinical trials were registered in the database of the US National Institutes of Health, and the number of new trials with MSC therapy is still growing exponentially (45). Until 2022, 416 published MSC-based clinical trials have evaluated MSCs’ effectiveness in treating various diseases (46). Some of the diseases that MSCs have been utilized to treat in clinical trials are summarized as follows (47). Cardiovascular diseases: cardiomyopathy, chronic heart failure, myocardial infarction and atherosclerotic plaque. Neurological diseases: hypoxic-ischemic brain lesions, Parkinson’s disease, stroke and Alzeheimer’s disease. Orthopedic diseases: osteochondral defects and osteoarthritis. Rheumatologic diseases: RA, ankylosing spondylitis, systemic sclerosis, lupus erythematosus, polymyositis and dermatomyositis and Sjögren’s syndrome. Endocrine diseases: Type 1 diabetes mellitus. Increasing investigations, regardless of animal studies or clinical trials, offer considerable evidence that MSCs transplantation is promising immunotherapy for autoimmune or systemic diseases.

In addition, the immune microenvironment has a particular impact on the occurrence, development, and metastasis of tumors, which are potential targets of MSCs in oncology (48). MSCs can be polarized by TLR signaling into two homogenously acting phenotypes, including pro-inflammatory MSC1 and anti-inflammatory MSC2 (49). Furthermore, MSC2 supported cancer growth and spread while MSC1 had anti-tumor effects (50). MSC1/MSC2 polarization is a convenient way to define a heterogeneous population of cells that may help in the further studies, as well as provides important guidance in the improvement of MSC-based therapies (49). Hashemi Seyed Mahmoud, considered a contributive scientist in this field, reported that MSCs isolated from breast cancer and normal breast adipose tissues shared similar morphology and immunophenotype but exhibited a diverse profile of immunoregulatory capacities and inflammatory cytokines (51). Cluster analysis also suggested future research directions on the functional elements of MSCs and how they could be converted to clinical and therapeutic advantages.

#### Yellow cluster: The cell source of MSCs

Although BMMSCs are recognized as the classic and principal source of MSCs in most scientific investigations and preclinical studies, non-marrow tissue-derived MSCs are identified in almost all adult tissues (52). Analysis of this cluster revealed that most MSC immunomodulation studies still focused on BMMSCs while exploring MSCs isolated from other tissues, including adipose tissue, umbilical cord, umbilical cord blood, placenta, and dental tissue. Numerous comparative studies have demonstrated heterogeneity of cytokine profiles, immunomodulatory effects, cell proliferation, and differentiation among different tissue-derived MSCs (53–58). Standardized studies on *in vitro* culture systems and stem cell sources will guide more precise clinical applications of MSCs in the future.

Based on the overlap analysis of keyword prevalence over time, research directions were monitored, and emerging hotspots were determined. The color of the node from blue to red represents the average publication year of keywords from 2012 to 2022. According to the results, exosomes, extracellular vesicles, secretion, extracellular matrix, scaffold, polarization, hypoxia, and inflammation were colored red. These keywords are the most popular topics in the field of MSC immunomodulation. Studies on MSC-based immunotherapy have shifted from cell therapy to cell-free therapy, which is a promising application in future medicine. Furthermore, studies exploring the mechanisms and effects of MSC immunomodulation remain the focus of this field.

As a bibliometric analysis, this study had several limitations. First, one database could not identify all studies. This study analyzed publications included in the WoSCC, which might represent the main research in the discipline. Second, only English literature was retrieved and included in this study; therefore, some important studies in other languages could have been omitted. Third, some recently published high-quality papers might have been omitted due to low citation frequencies shortly after publication. Therefore, the latest primary studies and non-English literature should be explored in future research.

## Conclusions

This study reveals global trends in MSC immunomodulation, which indicates that the immunomodulatory properties of MSCs have important research value and clinical application prospects. Since the 21st century, the total number of published studies has steadily increased, and China and the US are the two most productive regions in the world. *Stem Cell Research & Therapy* has the most publications in this field. Furthermore, the focus of studies has gradually shifted from “immunoregulatory effects” to “underlying mechanisms and immunotherapy”, indicating that further research might be focused on exploring the therapeutic potential of MSC immunomodulation. In particular, “exosomes”, “extracellular vesicles”, “secretion”, “polarization”, and “autophagy” are the recent promising hotspots.

## Author contributions

WZ: Data collection, data analysis and interpretation, final approval of manuscript. SY: Data collection, data analysis and interpretation. TX: Data analysis and interpretation, manuscript writing. SR: Data analysis and interpretation, manuscript writing. XY: Data collection, data analysis. ZY: Data collection, data analysis. LY: Conception and design, financial support, data analysis and interpretation, final approval of manuscript. All authors read and approved the final manuscript.

## Funding

This study was supported by the Key Research and Development Program of Liaoning Province (2021JH2/10300038 to LY).

## Conflict of interest

The authors declare that the research was conducted in the absence of any commercial or financial relationships that could be construed as a potential conflict of interest.

## Publisher’s note

All claims expressed in this article are solely those of the authors and do not necessarily represent those of their affiliated organizations, or those of the publisher, the editors and the reviewers. Any product that may be evaluated in this article, or claim that may be made by its manufacturer, is not guaranteed or endorsed by the publisher.

## Abbreviations

Mesenchymal stem cells, MSCs; bone marrow mesenchymal stem cells, BMMSCs; human leukocyte antigen DR isotype, HLA-DR; graft-versus-host disease, GVHD; rheumatoid arthritis, RA; systemic lupus erythematosus, SLE; inflammatory bowel disease, IBD; Web of Science Core Collection, WoSCC.
