# Supplementary material for: Global scientific trends on the immunomodulation of mesenchymal stem cells in the 21st century: A bibliometric and visualized analysis
Source: Front Immunol. 2022 Aug 24;13:984984. doi: 10.3389/fimmu.2022.984984 (PMC9449834; doi:10.3389/fimmu.2022.984984)
Supplement: Supplementary file 1 [file Table_1.docx]

## Supplementary Table 1. 111 high-frequency keywords concerning the immunomodulation of MSCs.

| **Cluster** | **Keywords** | **Counts** | **Average publication year** |
| --- | --- | --- | --- |
| 1 | mesenchymal stem cell | 2567 | 2016.47 |
| 1 | stem cell | 1995 | 2016.42 |
| 1 | differentiation | 904 | 2015.94 |
| 1 | immunomodulation | 861 | 2017.29 |
| 1 | inflammation | 440 | 2017.68 |
| 1 | macrophage | 266 | 2017.94 |
| 1 | cytokine | 263 | 2016.89 |
| 1 | mechanisms | 239 | 2017.33 |
| 1 | tissue regeneration | 202 | 2016.61 |
| 1 | tissue repair | 201 | 2015.88 |
| 1 | exosome | 197 | 2019.29 |
| 1 | survival | 187 | 2016.30 |
| 1 | extracellular vesicles | 159 | 2019.15 |
| 1 | tumor necrosis factor | 158 | 2016.16 |
| 1 | angiogenesis | 154 | 2017.64 |
| 1 | gene expression | 154 | 2016.31 |
| 1 | apoptosis | 142 | 2016.20 |
| 1 | Injury | 133 | 2016.89 |
| 1 | growth factors | 123 | 2015.82 |
| 1 | regenerative medicine | 119 | 2017.22 |
| 1 | secretion | 114 | 2018.18 |
| 1 | bone | 108 | 2015.87 |
| 1 | osteogenic differentiation | 91 | 2017.45 |
| 1 | osteogenesis | 87 | 2017.68 |
| 1 | oxidative stress | 71 | 2017.52 |
| 1 | senescence | 70 | 2016.74 |
| 1 | nf-kappa b | 69 | 2016.49 |
| 1 | interleukin-6 | 62 | 2016.00 |
| 1 | macrophage polarization | 62 | 2019.15 |
| 1 | polarization | 61 | 2018.56 |
| 1 | scaffold | 60 | 2018.45 |
| 1 | monocyte | 55 | 2016.60 |
| 1 | endothelial cell | 52 | 2015.52 |
| 1 | fibrosis | 52 | 2016.85 |
| 1 | fibroblasts | 50 | 2015.52 |
| 1 | conditioned medium | 49 | 2017.86 |
| 1 | extracellular matrix | 48 | 2017.46 |
| 1 | biomaterials | 47 | 2018.36 |
| 1 | lung | 44 | 2016.36 |
| 1 | hypoxia | 43 | 2017.86 |
| 1 | microrna | 43 | 2018.21 |
| 1 | hepatocyte growth-factor | 41 | 2016.12 |
| 1 | autophagy | 40 | 2018.15 |
| 1 | hydrogel | 40 | 2018.28 |
| 2 | proliferation | 821 | 2014.83 |
| 2 | immunosuppression | 531 | 2014.97 |
| 2 | t cell | 464 | 2015.15 |
| 2 | graft versus host disease | 422 | 2014.00 |
| 2 | inhibit | 406 | 2013.96 |
| 2 | interferon-gamma | 374 | 2015.42 |
| 2 | regulatory t cell | 327 | 2015.85 |
| 2 | dendritic cell | 271 | 2014.26 |
| 2 | in vivo | 215 | 2014.67 |
| 2 | suppression | 175 | 2015.40 |
| 2 | induction | 156 | 2016.12 |
| 2 | tolerance | 128 | 2014.70 |
| 2 | indoleamine 2,3-dioxygenase | 118 | 2015.18 |
| 2 | lymphocyte | 113 | 2015.73 |
| 2 | migration | 113 | 2016.65 |
| 2 | transforming growth factor-β | 96 | 2015.63 |
| 2 | prostaglandin e2 | 89 | 2015.67 |
| 2 | natural killer cell | 88 | 2014.83 |
| 2 | nitric oxide | 84 | 2014.79 |
| 2 | interleukin-10 | 79 | 2016.72 |
| 2 | th17 | 76 | 2016.80 |
| 2 | toll-like receptor | 72 | 2016.26 |
| 2 | immunoregulation | 69 | 2016.86 |
| 2 | microenvironment | 56 | 2016.68 |
| 2 | chemokine | 51 | 2015.69 |
| 2 | resistant | 51 | 2016.16 |
| 2 | autoimmune | 47 | 2016.74 |
| 2 | maturation | 46 | 2013.09 |
| 2 | cytotoxicity | 42 | 2017.05 |
| 2 | kidney transplantation | 42 | 2015.14 |
| 3 | transplantation | 787 | 2015.15 |
| 3 | therapy | 605 | 2016.82 |
| 3 | cell therapy | 296 | 2016.95 |
| 3 | arthritis | 199 | 2016.70 |
| 3 | model | 163 | 2016.64 |
| 3 | immune response | 157 | 2016.11 |
| 3 | immunotherapy | 155 | 2016.36 |
| 3 | cancer | 145 | 2016.26 |
| 3 | experimental autoimmune encephalomyelitis | 112 | 2014.54 |
| 3 | multiple sclerosis | 112 | 2015.25 |
| 3 | myocardial infarction | 89 | 2015.10 |
| 3 | inflammatory bowel disease | 78 | 2016.79 |
| 3 | pathogenesis | 67 | 2017.40 |
| 3 | clinical trial | 66 | 2017.12 |
| 3 | crohn's disease | 55 | 2016.22 |
| 3 | neuroprotection | 55 | 2015.38 |
| 3 | acute lung injury | 54 | 2017.87 |
| 3 | brain | 52 | 2015.73 |
| 3 | gene therapy | 52 | 2013.60 |
| 3 | animal model | 49 | 2016.61 |
| 3 | systemic lupus erythematosus | 48 | 2014.96 |
| 3 | central-nervous-system | 47 | 2015.11 |
| 3 | spinal cord injury | 46 | 2017.02 |
| 3 | sepsis | 45 | 2017.69 |
| 4 | bone marrow | 801 | 2015.46 |
| 4 | in vitro | 652 | 2014.94 |
| 4 | progenitor cell | 316 | 2014.50 |
| 4 | marrow stromal cells | 306 | 2012.49 |
| 4 | adipose tissue | 225 | 2016.02 |
| 4 | umbilical cord blood | 160 | 2014.93 |
| 4 | umbilical cord | 130 | 2016.39 |
| 4 | culture | 116 | 2015.62 |
| 4 | engraftment | 82 | 2013.16 |
| 4 | immunogenicity | 75 | 2015.24 |
| 4 | placenta | 73 | 2015.18 |
| 4 | bone marrow mesenchymal stem cells | 51 | 2016.98 |
